# Does Antibiotic Use Contribute to Biofilm Resistance in Sink Drains? A Case Study from Four German Hospital Wards

**DOI:** 10.3390/antibiotics13121148

**Published:** 2024-12-01

**Authors:** Nicole van Leuven, Ralf Lucassen, Anna Dicks, Patrick Braß, André Lipski, Dirk P. Bockmühl

**Affiliations:** 1 Faculty of Life Sciences, Rhine-Waal University of Applied Sciences, Marie-Curie-Straße 1, 47533 Kleve, Germany; 2 Food Microbiology and Hygiene, University of Bonn, Friedrich-Hirzebruch-Allee 7, 53115 Bonn, Germany; lipski@uni-bonn.de; 3 COMBAT AMR Project Consortium; 4 Helios Klinikum Krefeld, Lutherplatz 40, 47805 Krefeld, Germany

**Keywords:** hospital, biofilm, antibiotic stewardship, resistance, MIC

## Abstract

**Backgound**. As biofilms are known to harbour (multi-)resistant species, their presence in health settings must be considered critical. Although there is evidence that bacteria spread from drains to the outside, there is still a lack of research data focusing on drain biofilms from hospitals. **Methods**. We sampled biofilms from various wards of Helios Hospital Krefeld (Germany), where comprehensive antibiotic consumption data were available. Biofilms were analysed by cell counting, isolation of relevant bacterial groups and genetic and phenotypical resistance parameters. Data were correlated with the prescribed antibiotics of the respective ward. Furthermore, an ex situ biofilm model was employed to investigate the influence of sub-inhibitory antibiotics on the bacterial community and the prevalence of class 1 integrons. **Results**. Our results show that every ward harboured medically relevant bacterial species. While no significant differences were found in cell counts, the median prevalence of the resistance marker gene *intI1* correlated with the amount of prescribed antibiotics. In contrast, phenotypical resistances showed no similar tendency. In addition, melting curve analysis data and changes in *intI1* prevalence show that the composition of the bacterial community shifted depending on the biofilm and antibiotic. **Conclusions**. To the best of our knowledge, our study is the first considering possible correlations between the consumption data of hospital wards and resistances in drain biofilms the way we did. Based on our results, we conclude that sub-inhibitory concentrations of antibiotics have no general effect on biofilms in terms of bacterial community shift and occurrence of antibiotic-resistant species. Amongst other things, the effect depends on the initial composition of the bacterial community, the antibiotic used and the intrinsic bacterial resistance, e.g., prevalence of class 1 integrons.

## 1. Introduction

Biofilms are complex communities adhered to surfaces and are embedded in a polymer matrix. As a result, individual cells have an altered growth behaviour, metabolism and gene transcription compared to planktonic cells [1,2]. By retaining water and nutrients, the extracellular polymer matrix consisting of polymers, lipids, proteins and (extracellular) DNA improves the availability of important growth factors [1]. The extracellular matrix can furthermore act as physical protection that improves general resistances against different biotic and abiotic factors like extreme temperatures, pH values, UV light, dehydration, desiccation and antimicrobial substances like antibiotics or disinfectants [3,4,5,6,7,8].

There are several approaches to grow and analyse biofilms and their reaction to antimicrobial substances like antibiotics under lab conditions. A distinction can be made between static (e.g., in multiwell culture plates) and dynamic biofilm models (e.g., drip flow biofilm reactor) [9,10]. Some models are even designed to simulate the U-bends of sinks [11]. Although dynamic biofilm models resemble more realistic growth conditions, biofilm formation is usually tested using single- or multi-species models [10]. Still, using these complex ex situ biofilms as a base for experiments is much less common.

When looking at ex situ biofilms of unknown compositions, usually 16S rDNA sequencing followed by the assignment of reads to taxonomic units is performed [12]. Screening methods such as denaturing gradient gel electrophoresis (DGGE) or high-resolution melt analysis (HRMA) provide an initial insight into the changes in the biofilm community without determining the composition of the biofilm in detail [12,13].

Since hospital-acquired infections (HAIs) are one of the main medical problems in industrialised countries, biofilm development in healthcare environments must be considered particularly critical, especially since more than half of HAIs are estimated to be biofilm associated [14]. Biofilms in hospitals are often found on medical equipment, instruments and moist surfaces [4]. In addition to medical devices, biofilms are also present in the sink traps of patient rooms, which pose a risk of infection, especially for immunocompromised patients. There are specific characteristics with regard to the structure of sinks and pipes that might enhance cross-contamination of bacteria and dispersion of water droplets [15]. Depending on the drainage rate, droplets from the sink are able to splash and carry bacteria up to 1 m in the environment of the sink [16]. Additionally, sink drains can become contaminated by the same biofilm through drainage pipes [17]. Hospital sinks were found to be a source for carbapenemase-producing *Enterobacteriaceae* and also to be one of the most common sampling sites to be contaminated with Gram-negatives producing extended-spectrum beta-lactamases [18,19]. More specifically, most nosocomial infections associated with sinks or sink drains are caused by *Pseudomonas aeruginosa*, *Acinetobacter baumanii* or *Klebsiella* species [15]. Under favourable growth conditions, other *Gammaproteobacteria* like *Escherichia coli* are able to colonise sink traps up to the strainer in only a few days. Once the biofilm reaches the strainer, the spread of water droplets to external surfaces is facilitated. Patients or medical staff might then come into contact with these contaminated surfaces [17]. Furthermore, spore-forming Bacilli can be transferred from contaminated sinks to the sink’s countertop as well as gowns and hands [20]. Although handwashing sinks cannot be regarded as the primary source of antibiotic-resistant bacteria, they deserve attention in terms of induced resistances and biofilms in general [15,21]. Leftover antibiotics have been detected before and may also be enriched these environments even after rinsing steps [22].

In addition to antibiotics, common surfactants contained in conventional body care products might be additional drivers for resistances in sinks [23]. Phenotypic resistant bacteria carrying multiple resistance genes were found at various sampling points (e.g., U-bends of washbasins, shower siphons, washing machines and dishwashers) in households before [24,25,26]. It seems likely that comparable sites in hospitals are impacted in the same way.

According to the World Health Organization, increasing resistances against antibiotics are becoming a relevant threat for human health in general [27]. Almost all of the bacteria mentioned above are prominently associated with deaths related to resistances [28], which can be genetically anchored or just occur phenotypically and transiently [6]. Within biofilms, interactions of various compounds such as glycoproteins or lipids enable the binding of antibiotics. This impedes further diffusion and thereby reduces their concentrations within the biofilm [6,29]. Likewise, the diffusion in a biofilm matrix is influenced by its molecular weight and chemical properties [7]. The high cell density of biofilms can also result in a lower ratio of substance per cell [29]. Especially sub-inhibitory concentrations of antibiotics can increase extracellular matrix production or even induce biofilm development [30,31]. In addition, the cell density and genetic diversity provide an ideal basis for horizontal gene transfer [7,31,32]. The role of integrons in this process is significant, as they possess the capacity to integrate gene cassettes and facilitate the transfer of resistance genes into plasmids and chromosomes [33]. Especially class 1 integrons carrying the integrase gene *intI1* are commonly found in clinical samples and have also been described as a marker for anthropogenic pollution and multi-resistances in domestic settings [26,33,34]. In some cases, the co-selection of different resistances can be attributed to integrons [35]. For example it is common for class 1 integrons to carry *sul1* and *qac*∆*E* genes, which code for resistances against sulfonamides and quaternary ammonium compounds [33]. This makes a co-selection of both more likely [26].

As the overuse or misuse of antibiotics is believed to be one of the main drivers for microbial resistances [27], antibiotic stewardship and collecting consumption data are becoming increasingly important. In Germany, antibiotic surveillance is gradually being integrated and has been mandatory for ambulant surgeries since 2011 [36,37]. For monitoring the consumption or prescription of antibiotics, measures such as defined daily doses (DDDs), prescribed daily doses (PDDs) or recommended daily doses (RDDs) [36,38] are used. The DDD describes the mean daily dose of an antibiotic necessary for adults for its main indication and is mainly a theoretical and analytical figure. The PDD and RDD represent more application-oriented measures for the statistical description of antibiotic monitoring. While the PDD describes the quantity of antibiotics actually prescribed, the RDD is defined by the amount of antibiotics recommended for therapy [39].

As there are few obligations for hospitals to publish data on antibiotic consumption, there are few data available specifically on correlations between antibiotic usage and the resistance data of biofilms from the same hospital or its wards. This study aims to investigate a possible correlation between the amount of prescribed antibiotics and the presence of resistant bacteria in sink drain biofilms. In addition, we use an ex situ biofilm model to assess the influence of sub-inhibitory concentrations of antibiotics on the bacterial community composition as well as the genotypic resistance of some of the collected biofilms.

## 2. Results

### 2.1. Bacterial Counts and Identified Bacteria from Drain Biofilms

**Figure 1:**
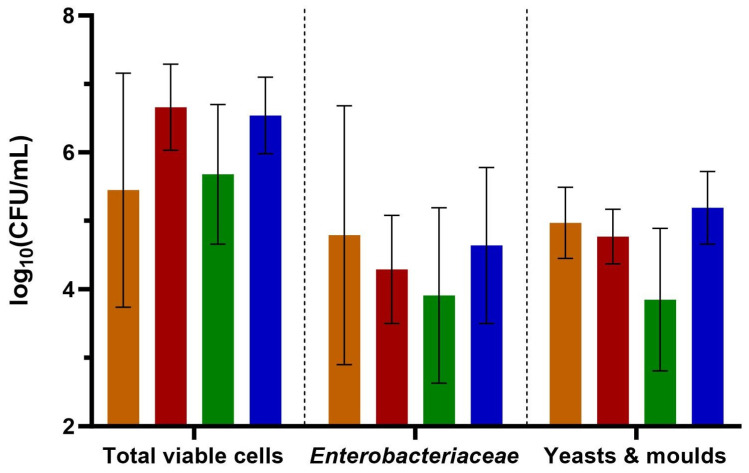
Mean cell counts of total viable cells, *Enterobacteriaceae* and yeasts and moulds in biofilm culture preparations from hospital wards A–D [log_10_(CFU/mL)]. Ward A: orange bars, ward B: red bars, ward C: green bars, ward D: blue bars. Whiskers represent standard deviations (*n* = 5). No statistically significant differences between wards were found using Tukey’s multiple comparison test. Detection limit of log_10_(CFU/mL) = 2.

Counts of total viable cells in biofilm culture preparations with PBS and glycerol varied between 10^5^ and 10^7^ CFU/mL, while counts for *Enterobacteriaceae* ranged between 10^3^ and 10^5^ CFU/mL (Figure 1). Values for yeasts and moulds were slightly higher for all wards except for ward C. No significant differences in mean cell counts between wards were found for each of the considered bacterial groups.

**Figure 2:**
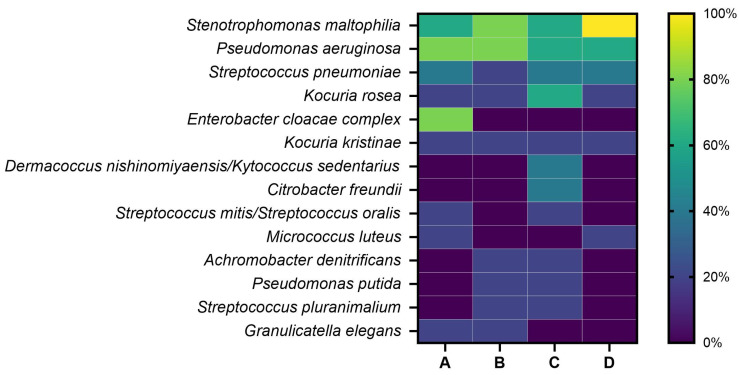
Occurrence [%] of bacterial isolates found in hospital wards A–D (*n* = 5 each). The colour gradient legend indicates the abundance of the isolate from high to low (0–100%). Only species found at least twice within all samples were considered for plotting. If the VITEK^®^ system was not able to distinguish isolates on a species level, suggested different species were used.

Preparation of isolates from different selective media was performed for identification of clinically relevant pathogens. Figure 2 depicts the abundance of bacterial species or groups which were found in the different hospital wards. Gram-positive and Gram-negative bacteria were identified. The most frequent species were *Stenotrophomonas maltophilia*, *Pseudomonas aeruginosa* and *Streptococcus pneumoniae.* Therefore, we chose these bacteria for the comparison of the mean MIC values of wards determined by E-tests and the summarised phenotypic resistance. *Kocuria rosea* was also found in all wards, but was not considered for phenotypical resistance testing since there is currently no VITEK^®^ AST card available for this species. Members of the *Enterobacter cloacae* complex were only found in ward A, although with a high abundance of 80%. Similarly, *Citrobacter freundii* and *Dermacoccus nishinonmiyaensis*/*Kytococcus sedentarius* were only isolated from ward C, but with an abundance of 60%.

### 2.2. Correlation of RDD and intI1 Prevalence as a Genetic Multi-Resistance Marker

**Figure 3:**
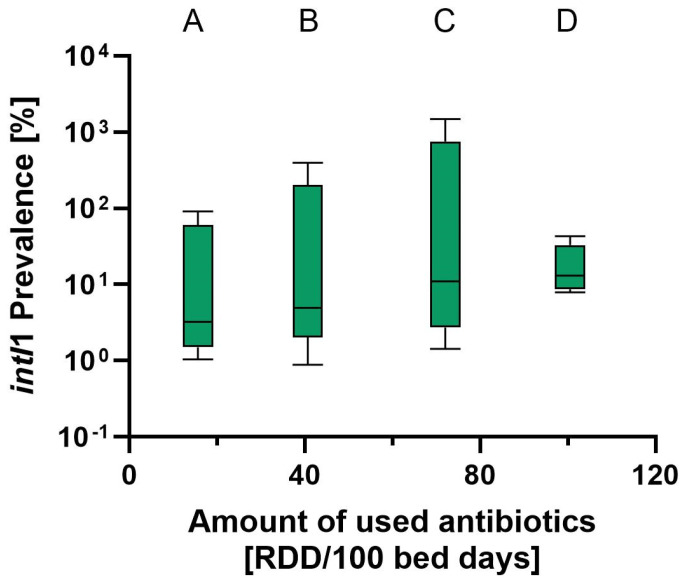
Median *intI1* prevalence [%] values in relation to the number of antibiotic prescriptions for hospital wards A–D. Lines show median values of each data set, boxes show 25th to 75th percentiles. Whiskers indicate the range of values from minimum to maximum.

For determination of genetically based resistances, we used the class 1 integron integrase gene (*intI1*) as a resistance marker and correlated *intI1* to the prescribed antibiotics. An increase in the median *intI1* value was found along with higher values for the RDD/100 bed days (Figure 3). Single *intI1* prevalences varied between about 10^0^ and 10^3^. The distribution of values within wards was relatively high, except for a very small range for ward D.

### 2.3. Correlation of RDD and Phenotypical Resistances

**Figure 4:**
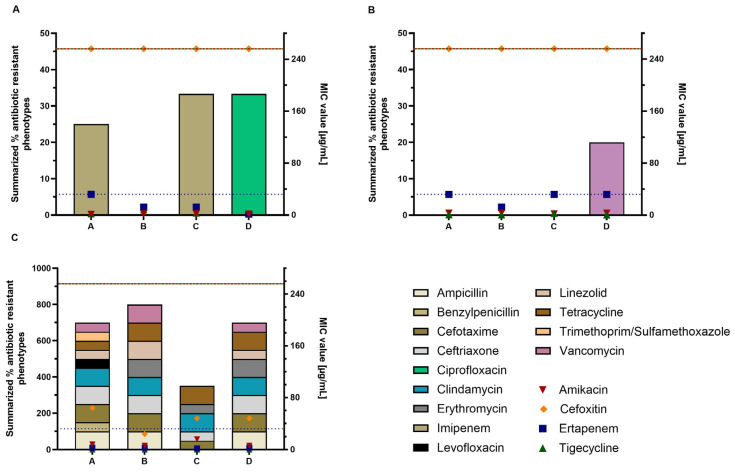
Phenotypic resistance data for isolates of *P. aeruginosa* (**A**), *S. maltophilia* (**B**) and *St. pneumoniae* (**C**) based on VITEK^®^ resistance data and E-testing from hospital wards A–D (*n* = 5 each). Stack bars plotted on the left y-axis show the sum of resistances against different antibiotics included in the respective VITEK^®^ AST cards [%]. Symbols plotted on the right y-axis indicate the mean MIC values of E-tests for different antibiotics [µg/mL]. Coloured lines indicate the maximum detection limits of the respective E-tests. AMK = Amikacin, CFX = Cefoxitin, ERT = Ertapenem, TGC = Tigecycline.

In addition to the genotypic resistance marker *intI1*, we determined the phenotypical resistance profiles of the most abundant bacterial species, *P. aeruginosa*, *S. maltophilia* and *St. pneumoniae*, using VITEK^®^ AST cards (bioMérieux, Marcy l’Étoile, France) and E-testing. The summarised percentages of resistant isolates identified by the VITEK^®^ system along with mean E-test results for amikacin, cefoxitin, ertapenem and tigecycline are plotted in Figure 4.

In contrast to what the summarised phenotypical resistance data for genetic resistances marker *intI1* indicate, there was no consistent tendency of increasing MIC values with increasing RDD values from ward A to D. However, the highest summarised value of the VITEK^®^ resistance data for two out of three bacteria was found in ward D. The results of other wards did not correlate. While for *P. aeruginosa* and *S. maltophilia* no resistances were found in ward B, the same ward showed the highest sum of resistant phenotypes for *St. pneumoniae*. Similarly, ward C along with ward D showed the highest resistance data for *P. aeruginosa*, but showed no resistances for *S. maltophilia* isolates and a low sum for *St. pneumoniae*.

Resembling the VITEK^®^ resistance data, no tendency of a correlation between increasing RDD and the MIC values of the E-tests for the wards was found. In relation to their MIC range, cefoxitin and ertapenem showed the highest MIC values, especially for *P. aeruginosa* and *S. maltophilia*.

### 2.4. Impact of Low-Dosed Antibiotics on the Microbial Communities in Complex Ex Situ Biofilms

**Figure 5:**
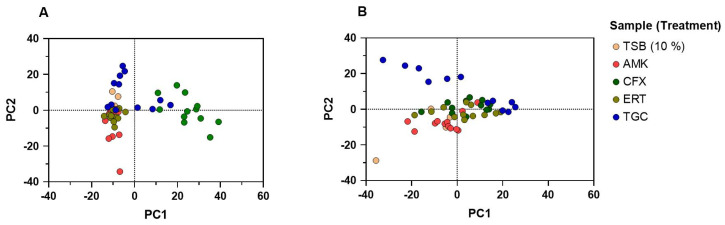
Principal component analysis of HRMA data for (**A**) biofilm D1 and (**B**) biofilm D2 for the growth control (TSB 10%) and treated with different antibiotics. Coloured dots show different biofilms and antibiotic treatments regardless of their concentration. Independent duplicates were performed. AMK = Amikacin, CFX = Cefoxitin, ERT = Ertapenem, TGC = Tigecycline.

Based on the MIC values found by E-testing, biofilms D1 and D2 were treated with four antibiotics in three different concentrations each. To evaluate shifts in the bacterial community due to the presence of the antibiotics, HRMA was performed. A principal component analysis (PCA) of HRMA data sorted by biofilm treatment can be seen in Figure 5. The respective melting curves of each data point can be found in Figures S1 and S2. Additional data from the PCA can be seen in Tables S2 and S3.

The PCA shows that especially ertapenem-treated samples cluster very well onto or near to the TSB controls for D1, implicating roughly similar biofilm communities. Amikacin- and tigecycline-treated samples are spread more widely around the control. Cefoxitin samples show the most noticeable scattered data points, strongly shifted to the right for D1. In contrast, for D2, no treatment group clusters that well. Yet every antibiotic shows some data points overlapping one of the controls, with the exception of the tigecycline-treated samples, suggesting the most different microbial communities.

**Figure 6:**
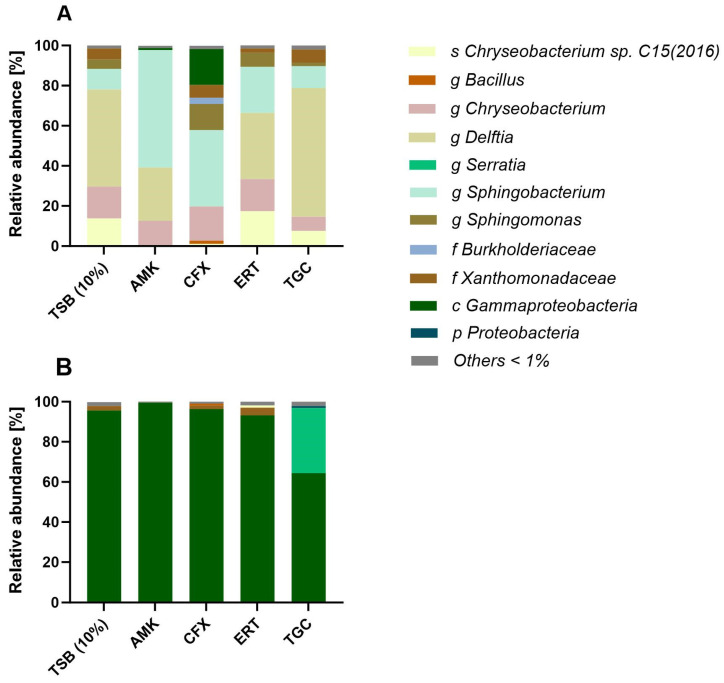
Relative abundances [%] of identified phylogenetic groups in the DNA of biofilm D1 (**A**) and D2 (**B**) for the untreated TSB (10%) growth control and treated with different antibiotics in 1 × MIC concentration. For duplicate testing, DNA was pooled to an equal amount of DNA for each sample. Prevalences lower than 1.0% are summarised as “Others”. AMK = Amikacin, CFX = Cefoxitin, ERT = Ertapenem, TGC = Tigecycline. s = species, g = genus, f = family, c = class and p = phylum.

Based on the HRMA data, selected DNA samples were sequenced and microbiome profiling for the *16S rDNA* was performed. The top hits of the read assignment for different phylogenetic levels can be seen in Figure 6.

The sequencing of biofilms showed big differences between the data sets of D1 and D2. For D1, mainly similar taxa were found. The most important groups found in all grown biofilms are represented by the genera *Chryseobacterium*, *Delftia* and *Sphingobacterium*. Especially *Chryseobacterium* sp. C15 (2016) was present in higher amounts in some of the biofilms. The abundances of these groups differed. In this data set, the cefoxitin treatment stands out visually, because no *Delftia* was found and there was a higher level of assignment to lower phylogenetic levels.

In contrast to the mostly very specific OTU assignments for D1, assignment to high phylogenetic groups was barely possible for the treated and untreated biofilms based on D2. With the exception of the tigecycline treatment, more than 90% were assigned to the class of *Gammaproteobacteria*, accompanied by low abundances of *Xanthomonadaceae*. After tigecycline treatment, the genus *Serratia* gained a larger share of about 30%. Looking at biofilm D1 on the class level (Figure S3) for better comparison shows the presence of 50% *Betaproteobacteria* along with about 30% of *Flavobacteriia* for the TSB control. After treatment, shares of *Alpha-*, *Beta*- and *Gammaproteobacteria* varied, while the classes of *Sphingobacteriia* and *Flavobacteriia* took in between 11% and 60%.

**Figure 7:**
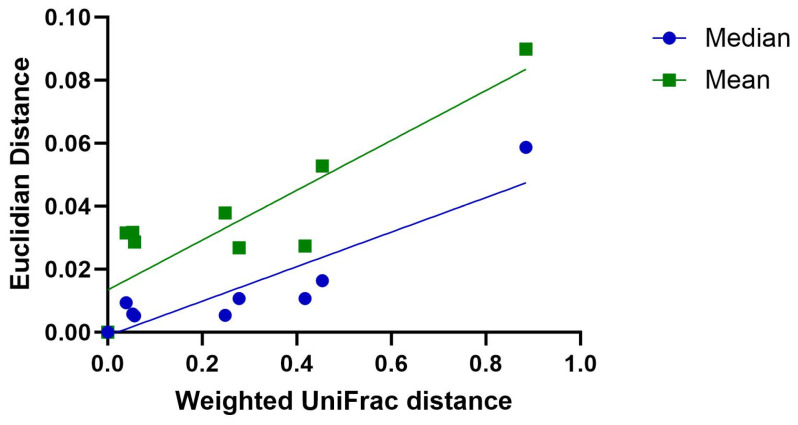
Correlation of mean and median Euclidian distances of HRMA data and weighted UniFrac distance of sequencing data from pairwise comparisons of treated biofilms with respective TSB (10%) growth controls. Lines show linear regressions. Coefficient of determination R^2^ equals 0.8222 and 0.7611, respectively.

For the assessment of a correlation between the HRMA and sequencing data, the weighted UniFrac distance was compared to the Euclidian distance of the melting curve data for all sample pairs (Figure 7).

Pairwise comparisons of TSB (10%) growth controls and biofilms with antibiotic treatment show a moderate-to-high correlation of the Euclidian distance of the HRMA data and the weighted UniFrac distance of the sequencing data for median and mean values.

**Figure 8:**
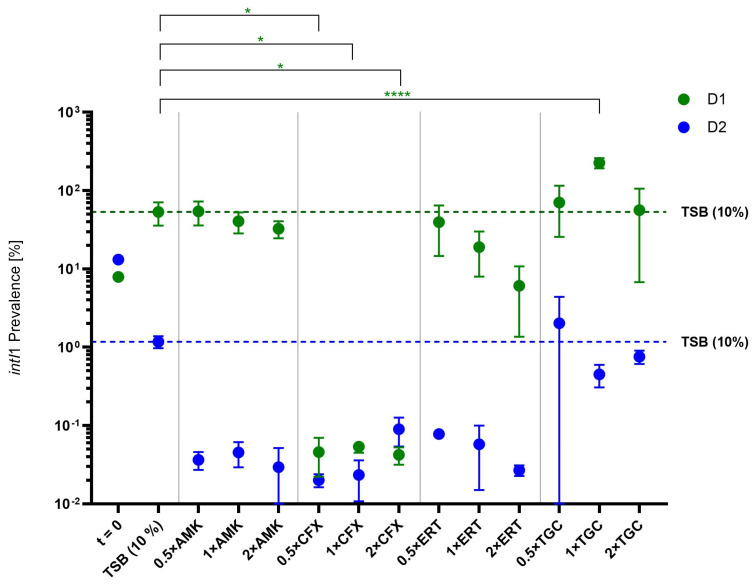
Mean *intI1* prevalences [%] of biofilms D1 and D2 for the original biofilm (t = 0) and growth controls (10% TSB) and after treatment with different antibiotics and concentrations. Bars represent standard deviations (*n* = 2). Dashed lines show mean values for growth controls in TSB (10%). Dunnett’s multiple comparisons test was used for determination of significant differences of values compared to the growth control (TSB 10%). Asterisks implicate statistical significances (* = *p* ≤ 0.05, **** = *p* ≤ 0.0001). AMK = Amikacin, CFX = Cefoxitin, ERT = Ertapenem, TGC = Tigecycline.

In addition to the determination of shifts in the bacterial community, the abundance of the *intI1* gene was investigated for the ex situ biofilms (Figure 8).

The data show that, although the *intI1* prevalences for the raw biofilm (t = 0) for both D1 and D2 are similar, they differ for the growth control and after treatments with antibiotics. For D1, the *intI1* prevalence increased during growth in TSB (10%) and amikacin and ertapenem treatment resulted in similar prevalences. Tigecycline values were slightly increased up to a highly significant change to a maximum of 226.1% (*p* ≤ 0.0001). Additionally, prevalences after treatment with cefoxitin were significantly lower (*p* < 0.05) and barely reached a value of 0.1%. Interestingly, values for D2 mostly decreased compared to the control and stayed below 0.1%, except for in the case of the tigecycline treatment, which led to similar values as for the control biofilms.

The stability controls of antibiotics were stable and showed inhibition zones with a maximum difference of 1 mm within the time span of 6 days of storage.

## 3. Discussion

### 3.1. Cell Counts and Biofilm Composition

Our results show that the composition and characteristics of biofilms collected from the drains of patient rooms can be very different. We found relatively high total cell counts based on high numbers of *Enterobacteriaceae*, yeasts and moulds in all drains. Since it takes fewer than 8 days to grow a biofilm with cell counts of more than 10^8^ CFU/cm^2^ in a drain trap simulation model [11], it is reasonable to find high cell counts in months- to years-old hospital U-bends as well. However, as we have no information about the age of the U-bend, the significance of cell counts in our case is negligible.

Since we used selective media for the isolation of bacterial groups, we created an intended bias towards finding especially clinically relevant and nosocomial pathogens against the overwhelming background of other environmental non-pathogenic bacteria. This way, we found clinically relevant and nosocomial pathogens in each ward, including species previously associated with sink-related hospital outbreaks [15]. Bacteria found in this study included *P. aeruginosa*, a significant pathogen present in sensitive medical settings, including intensive care units. It is also associated with persistent outbreaks of multidrug-resistant organisms (MDRO) [40]. Likewise, Gram-positive *St. pneumoniae* regularly causes outbreaks in hospitals. Crowded settings like communities and military settings can be affected too [41]. Additionally, *S. maltophilia*, which was present in every ward, is known as an emerging (nosocomial) pathogen and can be harmful, especially for immunocompromised patients [42,43]. Other bacteria like *Citrobacter freundii* or members of the *Enterobacter cloacae* complex were found frequently. Their abundance in only one ward supports the finding that nearby sinks can become contaminated with the same bacteria through back growth in the pipes [17].

In general, U-bends offer favourable conditions for biofilms due to their isolated, humid environment with a continuous nutrient supply [44]. Therefore, biofilm development must be assumed as inevitable. However, in order to reduce the colonisation of sink drains, special equipment to disinfect them is available. A secondary effect is the minimisation of the risk of outbreaks of MDROs, especially in hospitals [40]. Additionally, drain covers or usage of foam disinfectants instead of liquids might decrease biofilm growth and thereby infection risks [45].

### 3.2. Correlation of Resistance Data and Administrated Antibiotics

The presence of *Enterobacter*, *Citrobacter*, *Klebsiella*, *Pseudomonas* or *Stenotrophomonas* in the U-bend biofilms corresponded well with the relative abundance of class 1 integrons (*intI1*) found in our study. All of these genera have been associated with *intI1* in other studies as well [33]. The integrase gene *intI1* was chosen as a genetic marker for resistance because it proved to be a good proxy for genetic, but also phenotypical, resistances [26]. The presence of *intI1* can be treated as a marker not only for multi-resistances in drain biofilms, but also for anthropogenic pollution in general [26,34]. Compared to household samples, where the median *intI1* prevalence of sink biofilms was about 10^−1^ [26], our hospital samples showed higher median values between about 10^0^ and 10^1^. Naturally, hospital drains are more prone to contact with resistant bacteria and have putative contact with antibiotics. Increased handwashing and the related input of surfactants influence the *intI1* abundance as well [23]. Due to its higher resilience against outliers [46], we chose to use the median. The increasing median *intI1* values along with higher consumption of antibiotics suggest that high usage of antibiotics drives multi-resistances in patient room drains, although they are not directly prone to contact with antibiotics. However, when using the mean *intI1* prevalence, no such correlation could be seen.

In contrast to the genetic resistance marker, we found no clear trend of RDD values and the phenotypical resistances of isolates of the respective ward. The reason for that might be that the VITEK^®^2 compact system only allows resistance data to be obtained from isolates and not bacterial communities. Bacteria in biofilms are known to have an altered metabolism and higher resistance than individual planktonic cells [5]. The relevance of the resistance data collected here is therefore indicative in this context, but cannot be directly compared with the resistance behaviour of individual species in a biofilm. Conversely, when determining the prevalence of *intI1* in biofilms, the entire bacterial community is included in the results. This could explain these contradictory results. Nevertheless, Lucassen et al. (2019) were able to find a good correlation using a different analytical approach by grouping samples of similar *intI1* prevalences and correlating those groups with phenotypical resistances [26]. In addition to VITEK^®^ testing, we performed E-testing with four antibiotics that were not covered by the VITEK^®^ 2 compact analysis. Here, no correlation between MIC and RDD values was found either, presumably because of the previously mentioned reasons. A modified approach focusing not on specific antibiotics but on antibiotic groups might be useful in the future.

In summary, we found a high prevalence of class 1 integrons in the U-bends of hospital sink drains and found a reasonable number of clinically relevant bacteria carrying phenotypic resistances to multiple antibiotic classes. The prevalence of class 1 integrons could be positively correlated to the RDD values.

Although we focused on biofilms from whole hospital U-bends, other sample types like shower siphons or parts from toilets might be equally interesting. They might have even more direct contact with residual antibiotics from body surfaces or excretions. Biofilms from all three sample sites were confirmed to harbour the same typical genera, like *Acinetobacter*, *Citrobacter* or *Pseudomonas* [21]. Shower siphons have been identified as a hot spot for similar Gram-negative pathogens with several resistances and resistance genes in households too [24]. Yet patient sinks are an obvious place to look for resistant bacteria because the sinks might be misused by healthcare workers for disposal of patient wastewater or placement of contaminated patient materials [15]. Earlier findings confirm the presence of antibiotic residues in toilet, sink and shower drainpipes. Furthermore, residual sub-inhibitory amounts of antibiotics were detectable even after washing processes [21,22]. It also has to be considered that not only antibiotics but also surfactants present in many cleaning or body care products influence the community composition, and increase *intI1* prevalence and the probability of horizontal gene transfer in biofilms [23,47]. This highlights the importance of further investigations of biofilm samples from other sources in patient rooms. In this respect, dry surface biofilms also have to be considered [48].

### 3.3. Impact of Low-Dosed Antibiotics on the Microbial Communities in Complex Ex Situ Biofilms

To gain further insights into the influences of (sub-)inhibitory antibiotic concentrations on ex situ biofilms, we used biofilms from ward D (i.e., the ward with the highest RDD/100 bed days value). Since we only carried out a first screening, we decided on biofilms from this ward, although testing of the other wards might be equally interesting.

**Figure 9:**
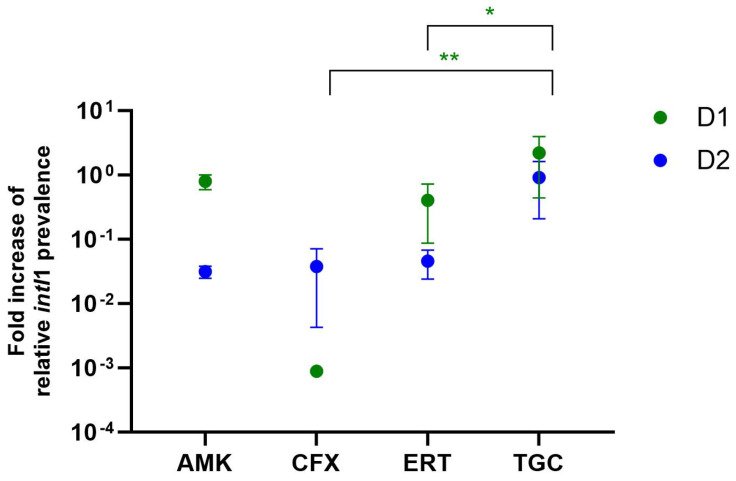
Mean fold increase in *intI1* prevalence for tested biofilms D1 and D2 treated with different antibiotics in 1 × MIC concentration. Tukey’s multiple comparisons test was used to look for significant differences in values compared to the growth control (TSB 10%). Asterisks implicate statistical significances (* = *p* ≤ 0.05, ** = *p* ≤ 0.01). AMK = Amikacin, CFX = Cefoxitin, ERT = Ertapenem, TGC = Tigecycline.

First, we tested how low antibiotic concentrations changed the *intI1* prevalence gene in the investigated biofilms. While prevalences mostly were similar to the control (D1) or even decreased (D2), Tigecycline treatment seemed to at least tendentially elevate the *intI1* prevalence. On the other hand, Cefoxitin treatment led to a decreased *intI1* prevalence for one of the biofilms. By not indicating a clear tendency, these findings are consistent with latest research showing that the fold increase in the *intI1* gene in biofilms varies based on the initial prevalence pre-treatment [23]. Our normalised results also show that different antibiotics lead to distinct *intI1* prevalences depending on the original biofilm (Figure 9).

The prominent shifts in community compositions observed in the sequencing results were also observed in the HRMA data, confirming earlier findings [13,49,50] and supporting the further use of HRMA as a screening tool for differences in biofilms. Similar correlations between those two types of data sets obtained by comparing the Euclidian distance and beta diversity measurements were shown before. Still, the Euclidian distance is only able to present quantitative statements about the (dis)similarities of biofilms. It does not allow any specific qualitative statement about the composition of a community, e.g., which bacterial genus or species is increased or decreased [13]. Using a similar method for single bacterial species, an identification accuracy of 95% can be achieved [51].

The community shifts were also accompanied by changes in *intI1* gene prevalence, which indicates that bacteria carrying class 1 integrons are either enriched or eliminated during the experiment. This is supported by the results for biofilm D2, where *intI1* prevalences mostly decreased (Figure 8). Here, it is likely that *intI1* carriers were eliminated. This matches the fact that most of the main bacteria carrying class 1 integrons are Gram-negatives [33], including multi-resistant ones, which are targeted by all of our tested antibiotics.

The antibiotics for the ex situ experiments were chosen based on E-testing. Single antibiotics were used in this study but further studies may use combinations of antibiotics to resemble a more clinical situation. For example, amikacin is often used for critical infections with Gram-negatives and commonly applied together with β-Lactam antibiotics [52]. Due to its broad-spectrum efficacy against polymicrobial infections, including both Gram-positive and Gram-negative bacteria [53], we used tigecycline in this study as well, although it is normally given intravenously [53,54]. Tigecycline’s broad-spectrum efficacy might also be the reason for the community changes, especially in biofilm D2. In this case, a relatively high share of *Serratia* spp. was present. Patient isolates of this genus have been found to show higher tigecycline resistances before and are a known carrier of class 1 integrons [33]. This could be the reason for the elevated *intI1* prevalence after tigecycline treatment.

## 4. Materials and Methods

### 4.1. Sampling and Sample Processing

Biofilm samples were obtained from patient room sinks at Helios Hospital Krefeld (Germany) in May and June 2023. Data on antibiotics prescriptions in 2022 were provided by the hospital in recommended daily doses (RDD) per 100 bed days. For each of the four wards (A–D), five biofilm samples (1–5) were collected. The total administration of antibiotics in these wards in 2022 is given in Table 1. Administration data from the last three years available (2020–2022) were relatively stable and can be seen in Table S1. Variations in values might have been caused by prophylactic and reasoned use for co-infections or secondary infections during the COVID-19 pandemic [55].

The complete U-bends of patient room sinks were removed and transferred to the lab immediately in sterile zip-bags. The biofilms inside the U-bends were scraped off with a sterile spatula and transferred into 50 mL tubes. One part of the biofilm was mixed with ten parts (*w*/*v*) of Phosphate-Buffered Saline (PBS; pH 7.4; Carl Roth GmbH + Co. KG, Karlsruhe, Germany) and filled with glycerol (≥99.5%, Carl Roth GmbH + Co. KG, Karlsruhe, Germany) to a final glycerol concentration of 25%. These cell suspensions, hereinafter referred to as culture preparations, were stored at −20 °C until further use [11]. Experiments were executed within the next four months to obtain culture preparation data, and additional experiments were executed within nine months (see Section 4.4). All experiments were performed at Rhine-Waal University, Kleve.

### 4.2. Cell Counts and Isolation of Relevant Bacteria

Determination of total viable counts, *Enterobacteriaceae* and yeast/moulds was carried out by plating the culture preparation described in Section 2.1 on Tryptic Soy Agar (TSA, Merck KGaA, Darmstadt, Germany), Mac Conkey Agar (Merck KGaA, Darmstadt, Germany) and Oxytetracycline Glucose Yeast Agar (OGYE, Thermo Fisher Scientific Inc., Waltham, MA, USA), respectively. A decimal serial dilution was prepared in PBS and 100 µL of each dilution was plated in duplicates on the corresponding media. Plates were incubated at 37 °C for 24 h (TSA, Mac Conkey Agar) or for 5 days at 25 °C (OGYE, Thermo Fisher Scientific Inc, Waltham, MA, USA). After incubation, plates with colony-forming unit (CFU) values between 10 and 300 were counted. The detection limit was therefore 100 CFU/mL. Consequently, all plates with colony counts were referred to as ≤100 CFU/mL. Mean log_10_ CFU/mL values of each culture preparation and hospital ward were calculated and plotted.

In addition to cell counts, additional selective media were used to isolate medically relevant bacterial groups from the biofilms. Columbia Agar with Colistin, nalidixic acid and 5% sheep blood (CNA, Thermo Fisher Scientific Inc., Waltham, MA, USA) was used to isolate Gram-positive bacteria, focusing on *Staphylococcus* spp. and *Streptococcus* spp. Xylose Lysin Desoxycholat Agar (XLD, Thermo Fisher Scientific Inc., Waltham, MA, USA) was used for the isolation of intestinal Gram-negative bacteria and *Pseudomonas*, and CFC Agar (CFC, Thermo Fisher Scientific Inc., Waltham, MA, USA) was used to single out *Pseudomonas* spp. One hundred microliters of dilutions were plated and incubated for 24–48 h at 37 °C (XLD, CNA) or 48 h at 25 °C (CFC). After incubation, optically distinguishable isolates were picked, subcultured on TSA (24 h at 37 °C) and analysed by the VITEK^®^ 2 compact system (bioMérieux, Marcy l’Étoile, France) or Epsilometer testing.

### 4.3. Resistance Analysis by VITEK^®^ 2 Compact and Epsilometer Testing

Isolates freshly subcultured on TSA were used for resistance analysis. For VITEK^®^ 2 compact analysis, cell suspensions and cards were prepared according to the manufacturer’s instructions (bioMérieux, Marcy l’Étoile, France). For identification, GN cards were used for isolates from CFC and XLD Agar. CNA isolates were identified using the GP ID card. The bacterial species or group with the highest assignment rate was chosen in the case of at least 80% probability. The resistance profile of Gram-negative bacteria was tested with the AST-N215 card. Depending on the identification of Gram-positive bacteria, AST-P592 or AST-ST03 (specifically for *Streptococcus* spp.) cards were used. *Pseudomonas aeruginosa (P. aeruginosa)*, *Stenotrophomonas maltophilia (S. maltophilia)* and *Streptococcus pneumoniae (St. pneumoniae)*, which were found in all wards, were used to compare the prevalence of the resistances of the different wards.

In addition to the VITEK^®^ results, a randomly chosen isolate of these bacteria from each ward was tested for amikacin (AMK), cefoxitin (CFX), ertapenem (ERT) and tigecycline (TGC) using Epsilometer tests (E-tests, bioMérieux, Marcy l’Étoile, France). These antibiotics belong to groups of antibiotics that are included in the provided consumption data but are not covered by the VITEK^®^ resistance cards. Tests were performed and analysed based on the European Committee on Antimicrobial Susceptibility Testing (EUCAST) breakpoints for antimicrobial susceptibility testing using the disk diffusion method [56]. A bacterial suspension in PBS of 0.5 McFarland was prepared and used for inoculation of Müller–Hinton Agar (Becton Dickinson GmbH, Heidelberg, Germany) with (*St. pneumoniae*) or without (*P. aeruginosa*, *S. maltophilia*) 5% horse blood and 20 mg/L β-nicotinamide adenine dinucleotide. E-tests were placed onto the agar plate with a sterile tweezer. Plates were incubated for 24 h at 37 °C. MIC values were read at the lowest concentration where the inhibition zone of the antibiotic intersected the MIC scale.

### 4.4. Impact of Low-Dosed Antibiotics During Biofilm Growth

To amplify homogeneous biofilm material for ex situ experiments, enrichments of two selected biofilms from ward D (D1 and D2) were prepared by application of 5 mL PBS/glycerol culture preparation to 45 mL TSB (10%, Merck KGaA, Darmstadt, Germany) followed by incubation for 3 days at room temperature [11]. In order to conduct a first screening, two biofilms from the same ward were chosen as independent duplicates influenced by similar types and amounts of antibiotics. We chose to use the samples from ward D with the highest RDD value to increase the visibility of possible changes in the *intI1* prevalence for both decreases and increases.

After incubation, glycerol was added to a concentration of 25% for consecutive storage at −20 °C. These enrichments were tested for the influence of sub-inhibitory concentrations of the antibiotics amikacin (AMK), cefoxitin (CFX), ertapenem (ERT) and tigecycline (TGC) in a simple 12-well cell culture plate setup. The selection of antibiotics was performed based on the groups of antibiotics that were included in the hospital’s consumption data. For each antibiotic, the lowest obtained MIC (determined by E-test; see Section 4.3), as well as half and double this concentration, was tested (Table 2).

As a growth medium, 10% TSB with or without the desired antibiotic concentrations was chosen. A 1.9 mL amount of media was inoculated with 100 µL of enrichment and was incubated at room temperature for 6 d including a media change after day 3. All experiments were executed in independent duplicates. On day 6, media were discarded and biofilms were washed with 2 mL PBS and 2D shaking (IKA™ ROCKER 2D DIGITAL; IKA-Werke GmbH & CO. KG, Staufen, Germany) for 30 s at 40 rpm twice to remove superficial cells. After discarding the washing solution, an additional 2 mL PBS and 1.5 g of borosilicate glass beads were applied to the wells. Cell extraction was performed by shaking the plate on a Thermomixer comfort (Eppendorf AG, Hamburg, Germany) for 5 min at 650 rpm. The complete volume of these cell suspensions was used for DNA extraction.

To prove the stability of the antibiotics at room temperature, disk diffusion tests based on EUCAST [56] were performed after 0, 3 and 6 d of storage. A cell suspension in PBS of 0.5 McFarland of *P. aeruginosa* and *Staphylococcus aureus* was streaked onto Müller–Hinton plates. Sterile disks were placed onto the media with sterile tweezers and inoculated with 25 µL of the antibiotic solution. Plates were incubated at 37 °C for 24 h followed by measurement of inhibition zone diameters.

### 4.5. DNA Extraction

Depending on the type of experiment, 2 mL of the culture preparation, the enrichment or the extracted cell suspension was used for DNA extraction with the Invitrogen™ PureLink™ Genomic DNA Mini Kit (Thermo Fisher Scientific Inc., Waltham, MA, USA). Elution was performed in two steps with 100 µL elution buffer each. DNA was stored at 4 °C for short-term storage and at −20 °C for long-term storage.

### 4.6. Determination of the Relative Prevalence (RP) of the Class 1 Integron Integrase Gene

To determine the relative prevalence of the class 1 integron integrase gene (*intI1*) per *16S rDNA* copy, standards with known copy numbers of these genes were prepared. Standards were obtained from a multi-resistant *Klebsiella pneumoniae* isolate and amplicons were checked for the correct length by gel electrophoresis.

As described by Lucassen et al., 1 µL of template DNA was applied to a mix of 5 µL FastStart Essential DNA Green Master (Roche Life Sciences, Mannheim, Germany), 4.8 µL PCR grade water and 0.1 µL each of forward and reverse primer (10 pmol). For *16S rDNA*, the primers F919 (5′-GAATTGACGGGGGCCCGCACAAG′3) and R1378 (5′CGGTGTGTACAAGGCCCGGGAACG′3) were used. For *intI1*, F165 (5′CGAACGAGTGGCGGAGGGTG′3) and R476 (5′TACCCGAGAGCTTGGCACCCA′3) were applied [26]. All PCR primers were purchased from Eurofins Genomics Germany GmbH, Ebersberg, Germany.

PCR was performed on a QuantStudio 3 qPCR device (Applied Biosystems by Thermo Fisher Scientific Inc., Waltham, MA, USA). The PCR program consisted of a pre-incubation (95 °C, 10 min), 35 cycles of denaturation (95 °C, 15 s), annealing (60 °C, 30 s) and elongation (72 °C, 60 s) and a final melting curve from 50 to 95 °C (0.1 °C/s). Based on the standard curves, copy numbers of the samples were calculated. In case of a negative PCR result, a 1:10 dilution was prepared and analysis was repeated. PCR-grade water acted as the non-template control. Samples were run in dependent duplicates for the raw biofilms and in independent multiple determinations for the ex situ experiments.

Determination of the relative prevalence of *intI1* [%] was conducted using the following equation:

Equation (1): Calculation of the relative prevalence (RP) of the *intI1* gene based on *16S rDNA* copies [26]:

(1) $$RP=\left(\frac{copies\;per\;µL\;of\;intI1}{copies\;per\;µL\;of\;16S\;rDNA}\right)\times 2.5\times 100$$

### 4.7. High-Resolution Melting Analysis (HRMA) Following Bacterial ITS Gene qPCR as a Screening Tool for Shift in Biofilm Communities

HRMA was performed following the approach of Andini et al. (2017) using the bacterial ITS sequence. Amplification was conducted using ITS1f (5′-TTGTACACACCGCCCG-′3) and ITS2r (5′-YGCCAAGGCATCCACC-′3) primer [51]. The template DNA (1.0 µL) was applied to a mix of 5.0 µL FastStart Essential DNA Green Master (Roche Deutschland Holding GmbH, Heidelberg, Germany), 4.8 µL PCR-grade water and 0.1 µL of 10 pmol primer. qPCR was performed using the following program: initial pre-incubation (300 s, 95 °C), 33 cycles of denaturation (95 °C, 30 s), annealing (55 °C, 30 s) and elongation (72 °C, 60 s). Immediately after the last PCR cycle, transition to melting analysis was performed by heating up to 95 °C for 28 s followed by cooling down to 28 °C for 30 s. Fluorescence was measured between 50 and 95 °C with a heating rate of 0,02 °C/s [13]. In case of a negative PCR result, a 1:10 dilution was prepared and analysis was repeated to avoid false-negative results based on interfering substances. PCR-grade water acted as a non-template control. DNA extracts were run in duplicates.

Normalised values of data between y = 0 and y = 1 for the active melting area of 75 to 95 °C were calculated. By using the negative derivation of fluorescence (−d(RFU)/dt) as a function of the temperature in °C, the melting data plots show melting points as peaks [57].

### 4.8. 16S rDNA Sequencing

The *16S rDNA* of the ex situ tested biofilms was sequenced to support the HRMA data and further investigate a possible shift in microbial communities. Since some concentrations of antibiotics were shown to have no significant influence on either the melting curves or *intI1* prevalence, we focused on the DNA of the growth controls and the 1 × MIC samples.

For independent duplicates, extracts were pooled by adding an equal amount of DNA of each. Sequencing of the bacterial *16S* V3-V4a region and the following microbiome profiling were conducted by an external service provider (Eurofins Genomics Europe Sequencing GmbH, Constance, Germany). The pipeline for data analysis consisted of Illumina chastity filtering, demultiplexing, primer clipping, merging and quality filtering of reads. These reads were used as the input for the microbiome profiling. Processing of OTUs and taxonomic assignment was conducted using QIIME software package version 1.9.1. For improvement of the results, abundances of bacterial taxonomic units were normalised using lineage-specific copy numbers of the relevant marker genes [58]. Results included processed reads, FASTA files, BIOM files, taxonomy tables, OTU tables and diversity statistics. Because the assignment of reads to high phylogenetic levels was barely possible for some biofilms, we decided to focus on the provided top-hit lists summarising different phylogenetic levels.

Since beta diversities determined by *16S rDNA* gene sequencing and the Euclidian distances of the HRMA data for pairwise comparisons were found to correlate previously [13], also in this study, the weighted UniFrac distance and the Euclidian distance were correlated. The mean melt curve data of duplicates were calculated and used to pairwise compare treated biofilms with the respective growth control in TSB (10%). The Euclidian distance from two melting curve data sets was calculated according to Equation (2).

Equation (2): Calculation of the Euclidian distance *d* [59]:

(2) $$d=\sqrt{{{(x}_{2}-x_{1})}^{2}+{{(y}_{2}-y_{1})}^{2}\;}$$

The mean and median values of the Euclidian distance data set were used for comparison with the weighted UniFrac distance.

### 4.9. Data Processing and Statistical Analysis

Statistical analysis of data was performed using GraphPad Prism v. 10.3.1 (GraphPad Software Inc., San Diego, CA, USA). To check for significant differences in cell counts or to compare results independent from controls, Tukey’s multiple comparison test was used. Dunnett’s multiple comparisons test was used to check for differences in *intI1* prevalences compared to the control biofilm. Correlations between the two parameters were checked for with simple linear regression.

## 5. Conclusions

The present study investigated the influence of residual antibiotics on hospital sink biofilms in situ and in an ex situ approach. There were barely any data available considering the possible correlations between antibiotic administration data and the different resistance factors of drain biofilms. We have now revealed a correlation between the median of the multi-resistances marker *intI1* gene of the biofilm gDNA and the amount of administrated antibiotics, although no comparable effect was observed for phenotypical resistances.

From experiments using ex situ biofilms in the presence of sub-inhibitory concentrations of antibiotics, we concluded that each combination of biofilm and antibiotic produces distinct changes regarding community shifts and (initial) *intI1* prevalence. However, for cefoxitin and tigecycline, the changes are more general and less distinct between biofilms. Testing more biofilms and other sample sites in patients’ rooms might extend this knowledge about the correlation between resistance data and the consumption of antibiotics and therefore comprise suitable next steps.

## Figures and Tables

**Table 1:** Properties of hospital wards A–D used for sampling for our experiments, including data on used antibiotics (RDD/100 bed days). Five biofilms from patient room sinks were sampled individually.

Ward	Included Specialties	Prescribed Antibiotics [RDD/100 Bed Days]
A	Cardiology	15.59
B	Orthopaedics, trauma and hand surgery	40.72
C	Urology, pneumology, gastroenterology, gynaecology	72.06
D	Plastic surgery, ophthalmology	100.51

**Table 2:** MIC ranges and used antibiotic concentrations for the 12-well experiments based on the lowest MIC value found in E-tests of different bacteria. AMK = Amikacin (MP Biomedicals Germany, Eschwege, Germany), CFX = Cefoxitin sodium (Apollo Scientific Ltd., Stockport, UK), ERT = Ertapenem sodium (MedChemExpress, Monmouth Junction, NJ, USA), TGC = Tigecycline (Thermo Fisher Scientific Inc., Waltham, MA, USA).

Antibiotic	MIC Range of E-Test [µg/mL]	0.5 × MIC [µg/mL]	1 × MIC [µg/mL]	2 × MIC [µg/mL]
AMK	0.016–256	0.5	1	2
CFX	0.016–256	0.8	1.6	3.2
ERT	0.002–32	0.375	0.75	1.5
TGC	0.016–256	0.008	0.016	0.032

## Data Availability

16S rRNA gene sequencing data are available at https://opus4.kobv.de/opus4-rhein-waal/frontdoor/index/index/docId/2082.

## Supplementary Materials

The following supporting information can be downloaded at https://www.mdpi.com/article/10.3390/antibiotics13121148/s1, Figure S1: Melting curves within the range of 75–95 °C of biofilm D1 in duplicates for (A) the original biofilm (t = 0),the enrichment, TSB controls, and for growth in different concentrations of Amikacin (B), Cefoxitin (C), Ertapenem (D) and Tigecycline (E); Figure S2: Melting curves within the range of 75–95 °C of biofilm D2 in duplicates for (A) the original biofilm (t = 0),the enrichment, TSB controls, and for growth in different concentrations of Amikacin (B), Cefoxitin (C), Ertapenem (D) and Tigecycline (E); Figure S3: Relative abundances [%] of phylogenetic classes in the DNA of biofilm D1 (A) and D2 (B) for the untreated TSB (10%) growth control and treated with different antibiotics in 1 × MIC concentration. For duplicate testing, DNA was pooled to an equal amount of DNA for each sample; Table S1: Properties of hospital wards A–D used for sampling for our experiments including data of used antibiotics (RDD/100 bed days) for the last three years available; Table S2: Additional data for the PCA shown in Figure 5A; Table S3: Additional data for the PCA shown in Figure 5B.
