# Supplementary material for: Does Antibiotic Use Contribute to Biofilm Resistance in Sink Drains? A Case Study from Four German Hospital Wards
Source: Antibiotics (Basel). 2024 Dec 1;13(12):1148. doi: 10.3390/antibiotics13121148 (PMC11672680; doi:10.3390/antibiotics13121148)
Supplement: Supplementary file 1 [file antibiotics-13-01148-s001.zip › antibiotics-3323904-supplementary.pdf]

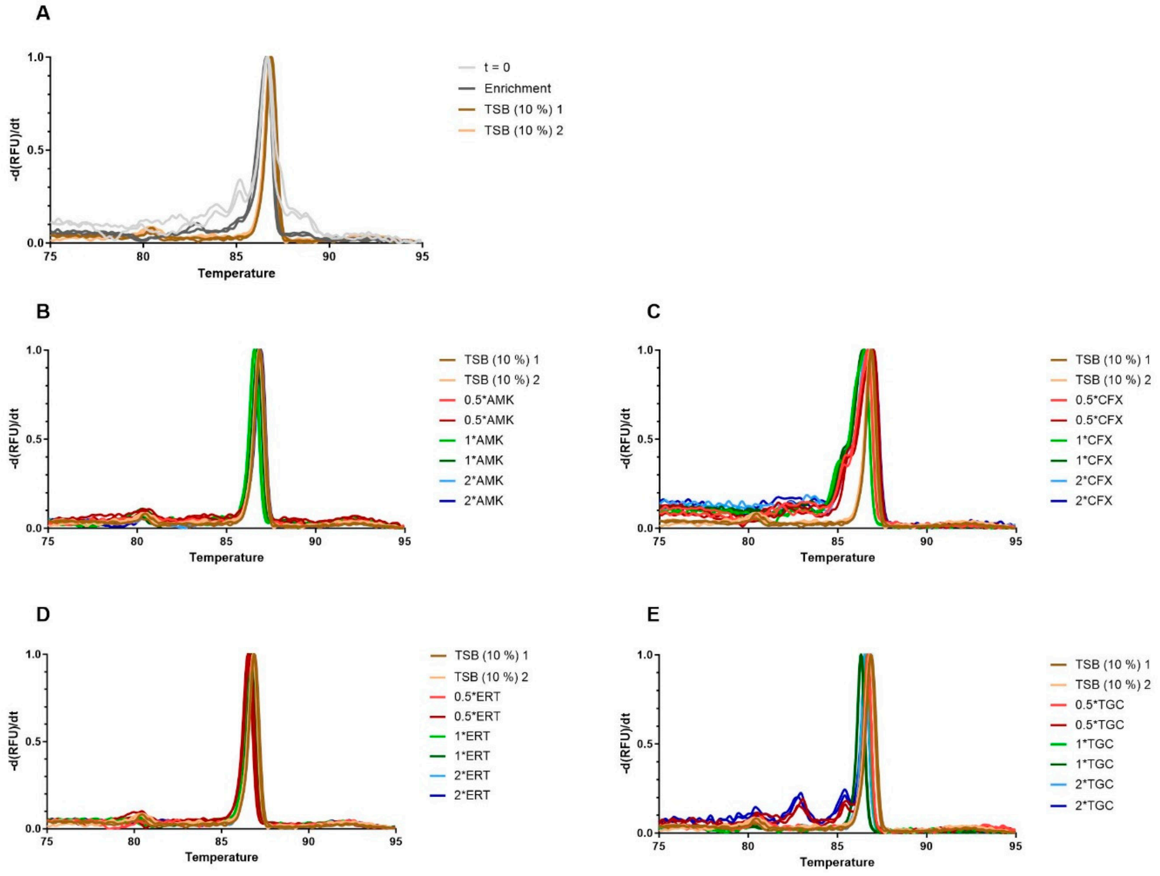

Figure S1: Melting curves within the range of 75-95 °C of biofilm D1 in duplicates for (A) the original biofilm (t = 0),the enrichment, TSB controls, and for growth in different concentrations of Amikacin (B), Cefoxitin (C), Ertapenem (D) and Tigecycline (E). Concentrations were based on the lowest MIC-value based on Epsilometer-tests to cover sub-inhibitory concentrations of antibiotics. Independent duplicates were done. Additionally, dependent duplicates of the same DNA extract was tested.

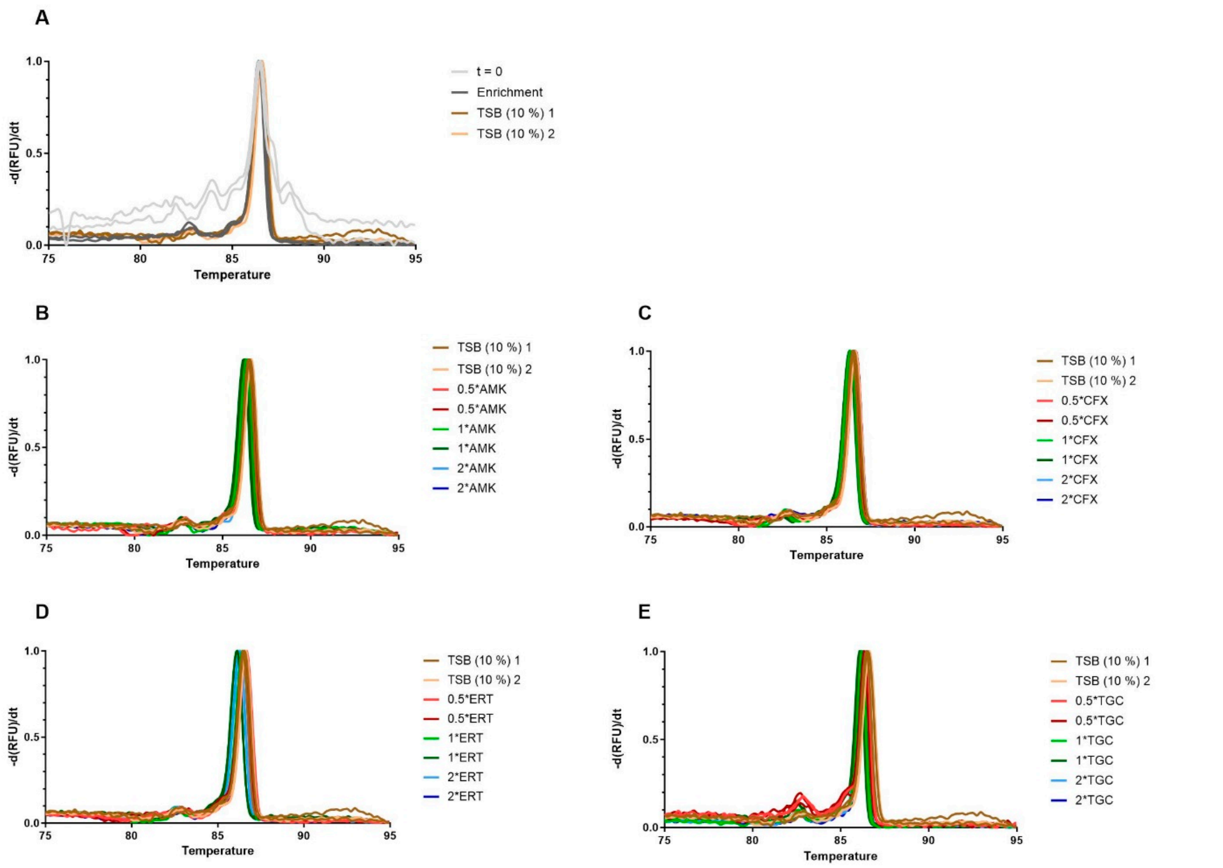

Figure S2: Melting curves within the range of 75-95 °C of biofilm D2 in duplicates for (A) the original biofilm (t = 0),the enrichment, TSB controls, and for growth in different concentrations of Amikacin (B), Cefoxitin (C), Ertapenem (D) and Tigecycline (E). Concentrations were based on the lowest MIC-value based on Epsilometer-tests to cover sub-inhibitory concentrations of antibiotics. Independent duplicates were done. Additionally, dependent duplicates of the same DNA extract was tested.

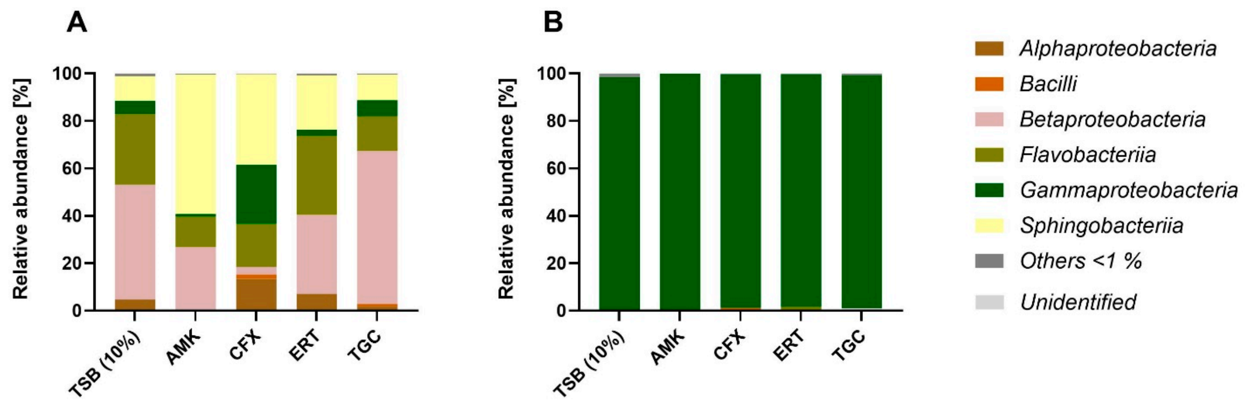

Figure S3: Relative abundances [%] of phylogenetic classes in the DNA of biofilm D1 (**A**) and D2 (**B**) for the untreated TSB (10%) growth control and treated with different antibiotics in 1 × MIC concentration. For duplicate testing, DNA was pooled to an equal amount of DNA for each sample. Prevalences lower

than 1.0% are summarised as "Others". AMK = Amikacin, CFX = Cefoxitin, ERT = Ertapenem, TGC = Tigecycline.

Table S1: Properties of hospital wards A-D used for sampling for our experiments including data of used antibiotics (RDD/100 bed days) for the last three years available. 5 biofilms of patient room sinks were sampled each.

Ward	Included specialties	Prescribed antibiotics [RDD/100 bed days]		
		2020	2021	2022
A	Cardiology	15.45	15.33	15.59
B	Orthopaedics, trauma and hand surgery	48.91	44.75	40.72
C	Urology, pneumology, gastroenterology, gynaecology	99.80	72.66	72.06
D	Plastic surgery, ophthalmology	97.89	101.29	100.51

Table S2: Additional data for the PCA shown in figure 5A

PC summary	PC1	PC2
Eigenvalue	237,9	97,39
Proportion of variance	50,62%	20,72%
Cumulative proportion of variance	50,62%	71,35%

Table S3: Additional data for the PCA shown in figure 5B

PC summary	PC1	PC2
Eigenvalue	191,4	96,82
Proportion of variance	40,72%	20,60%
Cumulative proportion of variance	40,72%	61,32%
